# A second monoclinic polymorph of {bis­[5-methyl-3-(trifluoro­meth­yl)pyrazol-1-yl]borato}{tris­[5-methyl-3-(trifluoro­meth­yl)pyrazol-1-yl]borato}cobalt(II): a structure containing a B—H⋯Co agostic inter­action

**DOI:** 10.1107/S1600536811021994

**Published:** 2011-06-11

**Authors:** Robert T. Stibrany, Joseph A. Potenza

**Affiliations:** a Department of Chemistry and Chemical Biology, Rutgers, The State University of New Jersey, 610 Taylor Road, Piscataway, New Jersey 08854 USA

## Abstract

The title compound, [Co(C_10_H_10_BF_6_N_4_)(C_15_H_13_BF_9_N_6_)], is a polymorph of the previously reported neutral cobalt(II) complex [Stibrany & Potenza (2010 ▶). *Acta Cryst.* E**66**, m506–m507], which contains one each of the monoanionic ligands, bis­[5-methyl-3-(trifluoro­meth­yl)pyrazol-1-yl]borate (Bp) and tris­[5-methyl-3-(trifluoro­meth­yl)pyrazol-1-yl]borate (Tp). A distorted octahedral coordination geometry of the Co^II^ atom results from ligation of an H atom, which is part of an agostic B—H⋯Co inter­action [H⋯Co = 2.12 (3) Å], and by five imine N atoms, two from a Bp ligand and three from a Tp ligand. Weak intra- and inter­molecular C—F⋯π inter­actions with F⋯centroid distances ranging from 3.025 (4) to 3.605 (4) Å are observed.

## Related literature

For our study of nitro­gen-containing heterocyles and their complexes with metal ions, see: Stibrany & Potenza (2006 ▶, 2009*a* ▶,*b* ▶); Stibrany *et al.* (1999 ▶, 2005 ▶, 2006 ▶). For a polymorph of the title compound, see: Stibrany & Potenza (2010 ▶). For oxidation studies of copper and cobalt complexes utilizing the title ligand, see: Gorun *et al.* (2000 ▶). For agostic inter­actions, see: Ruman *et al.* (2001 ▶, 2002 ▶); Siemer *et al.* (2001 ▶); Ghosh *et al.* (1998 ▶).
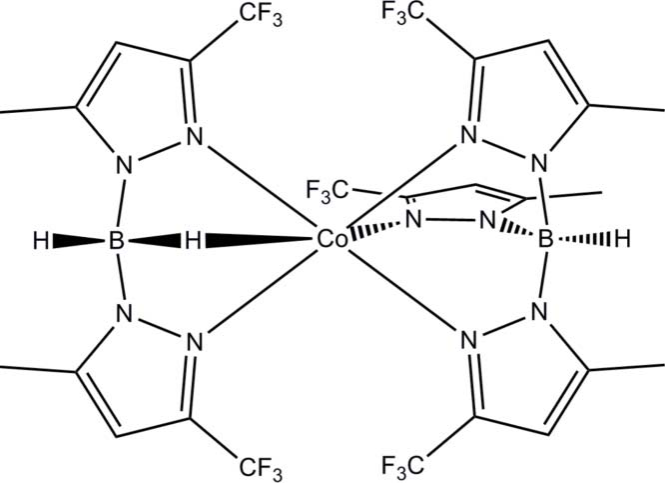


## Experimental

### 

#### Crystal data

[Co(C_10_H_10_BF_6_N_4_)(C_15_H_13_BF_9_N_6_)]
*M* *_r_* = 829.08
Monoclinic, 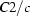

*a* = 18.593 (2) Å
*b* = 12.1167 (13) Å
*c* = 30.720 (3) Å
β = 102.721 (2)°
*V* = 6751.1 (13) Å^3^
*Z* = 8
Mo *K*α radiation
μ = 0.63 mm^−1^
*T* = 298 K
0.28 × 0.24 × 0.12 mm

#### Data collection

Bruker SMART CCD area-detector diffractometer
Absorption correction: multi-scan (*SADABS*; Blessing, 1995 ▶) *T*_min_ = 0.875, *T*_max_ = 1.00
32390 measured reflections
7431 independent reflections
5522 reflections with *I* > 2σ(*I*)
*R*_int_ = 0.032

#### Refinement

*R*[*F* ^2^ > 2σ(*F* ^2^)] = 0.059
*wR*(*F* ^2^) = 0.167
*S* = 1.00
7431 reflections
495 parameters
H atoms treated by a mixture of independent and constrained refinement
Δρ_max_ = 0.69 e Å^−3^
Δρ_min_ = −0.32 e Å^−3^

### 

Data collection: *SMART WNT/2000* (Bruker, 2000 ▶); cell refinement: *SAINT-Plus* (Bruker, 2000 ▶); data reduction: *SAINT-Plus*; program(s) used to solve structure: *SHELXS97* (Sheldrick, 2008 ▶); program(s) used to refine structure: *SHELXL97* (Sheldrick, 2008 ▶); molecular graphics: *ORTEPIII* (Burnett & Johnson, 1996 ▶) and *ORTEP-32* (Farrugia, 1997 ▶); software used to prepare material for publication: *SHELXTL* (Sheldrick, 2008 ▶) and *PLATON* (Spek, 2009 ▶).

## Supplementary Material

Crystal structure: contains datablock(s) I. DOI: 10.1107/S1600536811021994/rz2601sup1.cif

Structure factors: contains datablock(s) I. DOI: 10.1107/S1600536811021994/rz2601Isup2.hkl

Additional supplementary materials:  crystallographic information; 3D view; checkCIF report

## Figures and Tables

**Table 1:** Selected bond lengths (Å)

Co1—N7	2.100 (3)
Co1—H21*B*	2.12 (3)
Co1—N9	2.124 (3)
Co1—N3	2.124 (3)
Co1—N1	2.163 (3)
Co1—N5	2.172 (3)

**Table 2:** Flourine inter­action geometry (Å, °) *Cg*1, *Cg*2, *Cg*3 and *Cg*4 are the centroids of the N3/N4/C9/C8/C7, N5/N6/C14/C13/C12, N7/N8/C19/C18/C17 and N9/N10/C24/C23/C22 rings, respectively.

C—F⋯*Cg*	C—F	F⋯*Cg*	C⋯*Cg*	C—F⋯*Cg*
C1—F1⋯*Cg*4	1.333 (5)	3.319 (4)	3.798 (5)	100.7 (3)
C1—F2⋯*Cg*2^i^	1.331 (4)	3.341 (3)	4.565 (4)	152.6 (2)
C1—F3⋯*Cg*4	1.316 (5)	3.273 (3)	3.798 (5)	103.3 (3)
C11—F9⋯*Cg*3	1.295 (5)	3.025 (4)	3.826 (4)	119.1 (3)
C16—F10⋯*Cg*1	1.298 (5)	3.252 (4)	4.279 (4)	135.8 (3)
C16—F10⋯*Cg*2	1.298 (5)	3.208 (3)	4.061 (4)	122.8 (2)
C16—F11⋯*Cg*4^ii^	1.324 (5)	3.605 (4)	4.467 (4)	123.0 (3)
C21—F13⋯*Cg*1	1.321 (5)	3.373 (3)	4.346 (5)	130.3 (3)

Symmetry codes: (i) 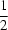 − *x*, 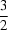 − *y*, −*z*; (ii) 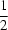 − *x*, 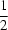 + *y*, 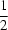 − *z*.

**Fig. 1.**
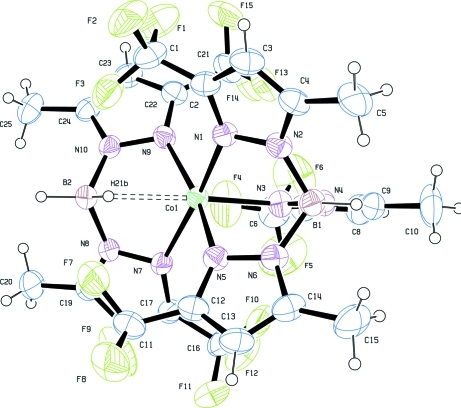
The molecular structure of the title compound showing displacement ellipsoids drawn at the 35% probability level. H atoms are shown as spheres of arbitrary radius.

## supplementary crystallographic information

### Comment

Our long-term interest in the synthesis and applications of nitrogen-containing heterocyles, such as expanded-ring imidazoles, and their complexes with metal ions (Stibrany & Potenza, 2009*a*) led us to prepare the title compound (I). With pyrazole, a variety of metal complexes has been prepared, predominantly with copper (Stibrany & Potenza, 2006), including the Cu(II) complex of an unusual (dimethylamino)methane bridged bis(pyrazole) ligand formed *via* a DMF aminalization (Stibrany *et al.*, 1999) and a novel binuclear µ-oxalato(1-benzylpyrazole)_2_(CF_3_SO_3_)_2_copper(II) compound resulting from the fixation of carbon dioxide (Stibrany *et al.*, 2005). A tris(pyrazolyl)arene ligand which forms geometrically constrained metal complexes has also been prepared (Stibrany *et al.*, 2006), as has a sterically strained trigonal-bipyramidal Cu(II) complex containing a 1-benzylpyrazole ligand (Stibrany & Potenza, 2009*b*). Copper and cobalt complexes utlizing the title ligand were prepared for oxidation studies (Gorun *et al.*, 2000).

Compound (I) (Fig. 1) contains a central Co(II) ion linked to a Tp and a Bp ligand. Ligation is effected by three imine N atoms of the Tp ligand, two imine N atoms of the Bp ligand, and an H atom which participates in a two-electron, three-center B—H···Co bond. The result is a distorted octahedral coordination geometry. Compound (I) is a polymorph of a previously reported structure of the same compound (II) (Stibrany & Potenza, 2010). The present polymorph was the result of a ligand displacement reaction in which a Tp ligand was added to a preformed Co(Bp)_2_ complex to form (I).

A number of cobalt(II) complexes containing mixed bis (Bp)- and tris pyrazolylborates (Tp) and C—H···Co agostic interactions have been reported, with H···Co distances ranging from 2.03 Å (Ruman *et al.*, 2002), 2.035 Å (Ruman *et al.*, 2001) to 2.334 Å (Siemer *et al.*, 2001). The H···Co distance in (I), 2.12 (3) Å, lies between these two extremes. Infrared spectral results also support the existence of an agostic 2-electron, 3-center bond in (I). B—H stretching vibrations for BH_2_ groups in which one of the H atoms is involved in a 2-electron, 3-center bond with a metal ion typically lie in the range 2100–2500 cm^-1^, with the lower value corresponding to the agostic interaction (Ghosh *et al.*, 1998). In (I), IR bands at 2570 and 2492 cm^-1^ are assigned as free B—H stretching vibrations, while the band at 2217 cm^-1^ is assigned to the bound B—H group.

The coordination geometries of both polymorphs are similar: in both, the largest deviations from octahedral symmetry are found with the H—Co—N angles associated with the bound hydrogen atom. The polymorphs differ fundamentally in the behavior of the trifluoromethyl groups. In the first polymorph, the molecules formed layers linked by intermolecular C—H···F hydrogen bonds in such a way as to form chains along the a cell direction. In contrast, the current polymorph exhibits no such hydrogen bonds; rather *PLATON* (Spek, 2009) reveals six intramolecular and two intermolecular C—F···*Cg* interactions (Table 2).

### Experimental

Both Tp and Bp ligands were prepared as previously reported (Gorun *et al*., 2000). To a flask containing 10 ml of acetonitrile, 60 mg of Co(Bp)_2_ (0.088 mmol) was disssolved to give a red-purple solution. Then 44 mg of KTp (0.088 mmol) was added and the mixture was allowed to stir for 10 min. giving little color change. The mixture was filtered to remove any solids and yielded a clear red-purple solution, which was left to evaporate slowly. Upon evaporation, a major dichroic red-orange phase (compound (I)) was separated mechanically and characterized. IR (KBr pellet, cm^-1^); 2570(Tp, B—H, w), 2492(Bp, B—H, w), 2217(Bp, B—H···Co, w), 1471(*s*), 1262(*s*), 1163(*s*), 1126(*s*), 1002(*s*), 800(*m*), 650(*m*).

### Refinement

Hydrogen atoms of the methyl groups were located on difference Fiourier maps and were restrained. H atoms of the pyrrole fragments were positioned geometrically using a riding model, with C—H = 0.98 Å for methyl H atoms, 0.95 Å for pyrrole H atoms, and with *U*_iso_(H) = 1.2 *U*_eq_(C). B—H hydrogen atom coordinates and isotropic displacement parameters were refined.

### Crystal data

**Table:**

[Co(C_10_H_10_BF_6_N_4_)(C_15_H_13_BF_9_N_6_)]	*F*(000) = 3320
*M**_r_* = 829.08	*D*_x_ = 1.631 Mg m^−^^3^
Monoclinic, *C*2/*c*	Mo *K*α radiation, λ = 0.71073 Å
Hall symbol: -C 2yc	Cell parameters from 910 reflections
*a* = 18.593 (2) Å	θ = 2.2–22.4°
*b* = 12.1167 (13) Å	µ = 0.63 mm^−^^1^
*c* = 30.720 (3) Å	*T* = 298 K
β = 102.721 (2)°	Prism, orange-red
*V* = 6751.1 (13) Å^3^	0.28 × 0.24 × 0.12 mm
*Z* = 8	

### Data collection

**Table:**

Bruker SMART CCD area-detector diffractometer	7431 independent reflections
Radiation source: fine-focus sealed tube	5522 reflections with *I* > 2σ(*I*)
graphite	*R*_int_ = 0.032
φ and ω scans	θ_max_ = 27.1°, θ_min_ = 2.1°
Absorption correction: multi-scan (*SADABS*; Blessing, 1995)	*h* = −23→23
*T*_min_ = 0.875, *T*_max_ = 1.00	*k* = −15→15
32390 measured reflections	*l* = −38→39

### Refinement

**Table:**

Refinement on *F*^2^	Primary atom site location: structure-invariant direct methods
Least-squares matrix: full	Secondary atom site location: difference Fourier map
*R*[*F*^2^ > 2σ(*F*^2^)] = 0.059	Hydrogen site location: inferred from neighbouring sites
*wR*(*F*^2^) = 0.167	H atoms treated by a mixture of independent and constrained refinement
*S* = 1.00	*w* = 1/[σ^2^(*F*_o_^2^) + (0.0985*P*)^2^ + 6.350*P*] where *P* = (*F*_o_^2^ + 2*F*_c_^2^)/3
7431 reflections	(Δ/σ)_max_ = 0.001
495 parameters	Δρ_max_ = 0.69 e Å^−^^3^
0 restraints	Δρ_min_ = −0.32 e Å^−^^3^

### Special details

**Table:**

Geometry. All e.s.d.'s (except the e.s.d. in the dihedral angle between two l.s. planes) are estimated using the full covariance matrix. The cell e.s.d.'s are taken into account individually in the estimation of e.s.d.'s in distances, angles and torsion angles; correlations between e.s.d.'s in cell parameters are only used when they are defined by crystal symmetry. An approximate (isotropic) treatment of cell e.s.d.'s is used for estimating e.s.d.'s involving l.s. planes.
Refinement. Refinement of *F*^2^ against ALL reflections. The weighted *R*-factor *wR* and goodness of fit *S* are based on *F*^2^, conventional *R*-factors *R* are based on *F*, with *F* set to zero for negative *F*^2^. The threshold expression of *F*^2^ > σ(*F*^2^) is used only for calculating *R*-factors(gt) *etc*. and is not relevant to the choice of reflections for refinement. *R*-factors based on *F*^2^ are statistically about twice as large as those based on *F*, and *R*- factors based on ALL data will be even larger.

### Fractional atomic coordinates and isotropic or equivalent isotropic displacement parameters (Å^2^)

**Table:**

	*x*	*y*	*z*	*U*_iso_*/*U*_eq_	
Co1	0.28653 (2)	0.88792 (3)	0.876253 (13)	0.04318 (14)	
F1	0.2096 (2)	1.0806 (3)	0.97276 (12)	0.1186 (11)	
F2	0.16548 (16)	0.9517 (3)	1.00620 (9)	0.1087 (10)	
F3	0.15337 (14)	0.9479 (3)	0.93573 (9)	0.1045 (9)	
F4	0.34590 (19)	1.0689 (3)	0.81275 (12)	0.1270 (12)	
F5	0.4369 (3)	1.0278 (4)	0.78800 (13)	0.178 (2)	
F6	0.4449 (3)	1.1573 (3)	0.83096 (17)	0.1721 (19)	
F7	0.15694 (15)	0.6605 (3)	0.85333 (12)	0.1158 (11)	
F8	0.18416 (17)	0.4977 (2)	0.84101 (12)	0.1135 (10)	
F9	0.1996 (2)	0.6240 (3)	0.79751 (10)	0.1371 (14)	
F10	0.38793 (14)	0.7600 (3)	0.81139 (9)	0.1108 (11)	
F11	0.33643 (17)	0.6704 (3)	0.75392 (12)	0.1122 (10)	
F12	0.38456 (17)	0.8212 (3)	0.74664 (13)	0.1245 (12)	
F13	0.36640 (15)	1.1157 (2)	0.93361 (10)	0.1001 (9)	
F14	0.36229 (18)	1.2527 (3)	0.89093 (12)	0.1275 (12)	
F15	0.3115 (2)	1.2626 (3)	0.94472 (13)	0.1412 (14)	
N1	0.30398 (14)	0.8830 (2)	0.94819 (8)	0.0483 (6)	
N2	0.36749 (14)	0.8317 (2)	0.96912 (8)	0.0500 (6)	
N3	0.40019 (15)	0.9216 (2)	0.88139 (9)	0.0533 (6)	
N4	0.44793 (14)	0.8664 (2)	0.91406 (9)	0.0520 (6)	
N5	0.30701 (14)	0.7122 (2)	0.88605 (9)	0.0488 (6)	
N6	0.37587 (15)	0.6891 (2)	0.91109 (8)	0.0509 (6)	
N7	0.25381 (13)	0.8603 (2)	0.80721 (8)	0.0482 (6)	
N8	0.18320 (13)	0.8959 (2)	0.79314 (9)	0.0502 (6)	
N9	0.24139 (13)	1.0495 (2)	0.87252 (8)	0.0483 (6)	
N10	0.17078 (13)	1.0446 (2)	0.84856 (8)	0.0476 (6)	
C1	0.2001 (2)	0.9716 (4)	0.97340 (14)	0.0728 (10)	
C2	0.2703 (2)	0.9112 (3)	0.98080 (11)	0.0546 (8)	
C3	0.3116 (2)	0.8782 (3)	1.02190 (12)	0.0673 (10)	
H3	0.2997	0.8882	1.0495	0.081*	
C4	0.3728 (2)	0.8286 (3)	1.01390 (11)	0.0605 (8)	
C5	0.4365 (3)	0.7800 (4)	1.04622 (13)	0.0868 (13)	
H5A	0.4474	0.7084	1.0360	0.130*	
H5B	0.4244	0.7733	1.0749	0.130*	
H5C	0.4788	0.8271	1.0486	0.130*	
C6	0.4145 (3)	1.0583 (4)	0.82312 (17)	0.0884 (13)	
C7	0.4429 (2)	0.9846 (3)	0.86181 (13)	0.0643 (9)	
C8	0.5164 (2)	0.9703 (4)	0.88129 (15)	0.0771 (11)	
H8	0.5564	1.0047	0.8734	0.093*	
C9	0.51825 (19)	0.8952 (3)	0.91462 (14)	0.0681 (10)	
C10	0.5832 (2)	0.8483 (6)	0.94721 (19)	0.1079 (18)	
H10A	0.5791	0.8656	0.9771	0.162*	
H10B	0.6278	0.8797	0.9418	0.162*	
H10C	0.5842	0.7697	0.9436	0.162*	
C11	0.2065 (2)	0.6009 (3)	0.83940 (14)	0.0686 (10)	
C12	0.2820 (2)	0.6161 (3)	0.86740 (11)	0.0561 (8)	
C13	0.3333 (2)	0.5337 (3)	0.87903 (13)	0.0668 (9)	
H13	0.3286	0.4603	0.8700	0.080*	
C14	0.3926 (2)	0.5815 (3)	0.90657 (12)	0.0630 (9)	
C15	0.4641 (3)	0.5313 (4)	0.92941 (17)	0.0940 (14)	
H15A	0.5040	0.5758	0.9241	0.141*	
H15B	0.4679	0.4583	0.9179	0.141*	
H15C	0.4665	0.5273	0.9609	0.141*	
C16	0.3460 (2)	0.7703 (4)	0.77177 (13)	0.0711 (10)	
C17	0.27437 (18)	0.8250 (3)	0.77033 (11)	0.0553 (8)	
C18	0.2182 (2)	0.8380 (3)	0.73323 (12)	0.0637 (9)	
H18	0.2188	0.8197	0.7039	0.076*	
C19	0.16104 (19)	0.8835 (3)	0.74851 (11)	0.0583 (8)	
C20	0.0860 (2)	0.9147 (4)	0.72326 (14)	0.0837 (13)	
H20A	0.0529	0.8539	0.7232	0.126*	
H20B	0.0878	0.9329	0.6931	0.126*	
H20C	0.0689	0.9775	0.7372	0.126*	
C21	0.3222 (2)	1.1949 (3)	0.91380 (15)	0.0767 (11)	
C22	0.25220 (19)	1.1548 (3)	0.88547 (11)	0.0553 (8)	
C23	0.1893 (2)	1.2165 (3)	0.86992 (13)	0.0632 (9)	
H23	0.1830	1.2915	0.8742	0.076*	
C24	0.13797 (19)	1.1441 (3)	0.84681 (11)	0.0546 (8)	
C25	0.0598 (2)	1.1627 (4)	0.82443 (15)	0.0764 (11)	
H25A	0.0505	1.1322	0.7949	0.115*	
H25B	0.0498	1.2405	0.8227	0.115*	
H25C	0.0284	1.1275	0.8412	0.115*	
B1	0.4190 (2)	0.7788 (3)	0.94208 (12)	0.0532 (8)	
B2	0.1443 (2)	0.9287 (3)	0.83098 (13)	0.0528 (8)	
H1B	0.4671 (19)	0.741 (3)	0.9652 (12)	0.060 (9)*	
H21B	0.1701 (18)	0.870 (3)	0.8601 (12)	0.055 (9)*	
H22B	0.085 (2)	0.929 (3)	0.8187 (12)	0.061 (9)*	

### Atomic displacement parameters (Å^2^)

**Table:**

	*U*^11^	*U*^22^	*U*^33^	*U*^12^	*U*^13^	*U*^23^
Co1	0.0438 (2)	0.0430 (2)	0.0410 (2)	0.00260 (16)	0.00549 (15)	0.00325 (16)
F1	0.143 (3)	0.0821 (18)	0.145 (3)	0.0403 (18)	0.063 (2)	0.0102 (18)
F2	0.1070 (19)	0.156 (3)	0.0809 (17)	0.0325 (19)	0.0585 (15)	0.0171 (17)
F3	0.0719 (15)	0.173 (3)	0.0713 (16)	0.0321 (17)	0.0217 (13)	−0.0014 (17)
F4	0.103 (2)	0.150 (3)	0.122 (3)	−0.007 (2)	0.0127 (19)	0.072 (2)
F5	0.238 (5)	0.208 (5)	0.111 (3)	0.051 (4)	0.089 (3)	0.060 (3)
F6	0.196 (4)	0.096 (2)	0.207 (5)	−0.045 (3)	0.007 (3)	0.055 (3)
F7	0.0792 (17)	0.095 (2)	0.161 (3)	−0.0026 (15)	0.0009 (18)	−0.042 (2)
F8	0.110 (2)	0.0618 (14)	0.163 (3)	−0.0306 (14)	0.018 (2)	−0.0054 (16)
F9	0.129 (3)	0.192 (4)	0.0765 (19)	−0.080 (2)	−0.0068 (17)	0.014 (2)
F10	0.0732 (15)	0.178 (3)	0.0759 (16)	0.0625 (18)	0.0036 (13)	−0.0179 (17)
F11	0.108 (2)	0.093 (2)	0.137 (3)	0.0313 (16)	0.0305 (18)	−0.0323 (18)
F12	0.100 (2)	0.135 (3)	0.162 (3)	0.0265 (19)	0.080 (2)	0.032 (2)
F13	0.0914 (17)	0.0696 (15)	0.113 (2)	−0.0057 (13)	−0.0352 (15)	−0.0066 (13)
F14	0.115 (2)	0.119 (2)	0.135 (3)	−0.054 (2)	−0.002 (2)	0.019 (2)
F15	0.136 (3)	0.120 (2)	0.144 (3)	0.008 (2)	−0.020 (2)	−0.084 (2)
N1	0.0522 (14)	0.0476 (13)	0.0450 (13)	0.0039 (11)	0.0104 (11)	0.0029 (11)
N2	0.0582 (15)	0.0491 (14)	0.0400 (13)	0.0015 (12)	0.0047 (11)	0.0040 (11)
N3	0.0535 (15)	0.0562 (15)	0.0500 (14)	−0.0023 (12)	0.0106 (12)	0.0088 (12)
N4	0.0429 (13)	0.0603 (16)	0.0512 (15)	−0.0009 (11)	0.0065 (11)	−0.0004 (12)
N5	0.0528 (14)	0.0433 (13)	0.0492 (14)	0.0012 (11)	0.0088 (11)	0.0015 (11)
N6	0.0626 (16)	0.0455 (14)	0.0437 (13)	0.0090 (12)	0.0097 (12)	0.0072 (11)
N7	0.0433 (13)	0.0543 (14)	0.0451 (13)	0.0074 (11)	0.0058 (10)	−0.0027 (11)
N8	0.0422 (13)	0.0571 (15)	0.0476 (14)	0.0037 (11)	0.0022 (10)	−0.0063 (11)
N9	0.0477 (13)	0.0472 (14)	0.0454 (13)	0.0040 (11)	0.0007 (11)	0.0004 (11)
N10	0.0440 (13)	0.0531 (14)	0.0448 (13)	0.0069 (11)	0.0076 (10)	0.0010 (11)
C1	0.083 (3)	0.081 (3)	0.062 (2)	0.013 (2)	0.034 (2)	0.001 (2)
C2	0.068 (2)	0.0515 (18)	0.0476 (17)	−0.0029 (15)	0.0201 (15)	−0.0016 (13)
C3	0.092 (3)	0.069 (2)	0.0425 (17)	0.002 (2)	0.0194 (17)	−0.0003 (16)
C4	0.077 (2)	0.058 (2)	0.0428 (17)	−0.0026 (17)	0.0049 (16)	−0.0010 (14)
C5	0.102 (3)	0.105 (3)	0.0439 (19)	0.014 (3)	−0.005 (2)	0.006 (2)
C6	0.095 (3)	0.085 (3)	0.095 (3)	−0.012 (3)	0.041 (3)	0.022 (3)
C7	0.068 (2)	0.063 (2)	0.065 (2)	−0.0081 (17)	0.0233 (17)	0.0013 (17)
C8	0.063 (2)	0.088 (3)	0.087 (3)	−0.020 (2)	0.032 (2)	−0.005 (2)
C9	0.0462 (18)	0.085 (3)	0.073 (2)	−0.0031 (17)	0.0143 (16)	−0.015 (2)
C10	0.042 (2)	0.167 (5)	0.107 (4)	−0.002 (3)	−0.001 (2)	0.011 (4)
C11	0.081 (3)	0.053 (2)	0.071 (2)	−0.0135 (18)	0.016 (2)	−0.0053 (17)
C12	0.075 (2)	0.0457 (17)	0.0487 (17)	−0.0050 (16)	0.0167 (16)	−0.0002 (14)
C13	0.093 (3)	0.0423 (17)	0.065 (2)	0.0041 (18)	0.017 (2)	−0.0015 (16)
C14	0.086 (3)	0.0514 (18)	0.0540 (19)	0.0207 (18)	0.0209 (18)	0.0124 (15)
C15	0.109 (3)	0.079 (3)	0.091 (3)	0.045 (3)	0.015 (3)	0.013 (2)
C16	0.069 (2)	0.084 (3)	0.064 (2)	0.020 (2)	0.0221 (19)	−0.001 (2)
C17	0.0560 (18)	0.060 (2)	0.0492 (17)	0.0090 (15)	0.0106 (14)	−0.0003 (15)
C18	0.068 (2)	0.075 (2)	0.0458 (18)	0.0087 (19)	0.0090 (16)	−0.0024 (16)
C19	0.0547 (18)	0.069 (2)	0.0460 (17)	0.0036 (16)	−0.0002 (14)	−0.0044 (15)
C20	0.062 (2)	0.115 (4)	0.063 (2)	0.016 (2)	−0.0099 (18)	−0.011 (2)
C21	0.085 (3)	0.050 (2)	0.085 (3)	−0.009 (2)	−0.003 (2)	−0.003 (2)
C22	0.067 (2)	0.0434 (16)	0.0527 (18)	0.0017 (15)	0.0069 (15)	0.0021 (14)
C23	0.077 (2)	0.0462 (17)	0.068 (2)	0.0150 (17)	0.0185 (18)	0.0057 (16)
C24	0.0571 (18)	0.0566 (18)	0.0516 (18)	0.0141 (15)	0.0153 (14)	0.0050 (14)
C25	0.060 (2)	0.084 (3)	0.087 (3)	0.023 (2)	0.020 (2)	0.017 (2)
B1	0.055 (2)	0.057 (2)	0.0445 (18)	0.0105 (17)	0.0045 (15)	0.0072 (16)
B2	0.0422 (18)	0.060 (2)	0.056 (2)	−0.0015 (16)	0.0101 (15)	−0.0080 (17)

### Geometric parameters (Å, °)

**Table:**

Co1—N7	2.100 (3)	C2—C3	1.384 (5)
Co1—H21B	2.12 (3)	C3—C4	1.356 (5)
Co1—N9	2.124 (3)	C3—H3	0.9300
Co1—N3	2.124 (3)	C4—C5	1.489 (5)
Co1—N1	2.163 (3)	C5—H5A	0.9600
Co1—N5	2.172 (3)	C5—H5B	0.9600
F1—C1	1.333 (5)	C5—H5C	0.9600
F2—C1	1.331 (4)	C6—C7	1.487 (6)
F3—C1	1.316 (5)	C7—C8	1.376 (6)
F4—C6	1.252 (6)	C8—C9	1.365 (6)
F5—C6	1.293 (6)	C8—H8	0.9300
F6—C6	1.325 (6)	C9—C10	1.500 (6)
F7—C11	1.315 (5)	C10—H10A	0.9600
F8—C11	1.322 (4)	C10—H10B	0.9600
F9—C11	1.295 (5)	C10—H10C	0.9600
F10—C16	1.298 (5)	C11—C12	1.487 (5)
F11—C16	1.324 (5)	C12—C13	1.373 (5)
F12—C16	1.317 (5)	C13—C14	1.363 (6)
F13—C21	1.321 (5)	C13—H13	0.9300
F14—C21	1.331 (5)	C14—C15	1.490 (6)
F15—C21	1.303 (5)	C15—H15A	0.9600
N1—C2	1.337 (4)	C15—H15B	0.9600
N1—N2	1.364 (4)	C15—H15C	0.9600
N2—C4	1.358 (4)	C16—C17	1.480 (5)
N2—B1	1.538 (5)	C17—C18	1.375 (5)
N3—C7	1.337 (4)	C18—C19	1.368 (5)
N3—N4	1.360 (4)	C18—H18	0.9300
N4—C9	1.350 (4)	C19—C20	1.488 (5)
N4—B1	1.538 (5)	C20—H20A	0.9600
N5—C12	1.335 (4)	C20—H20B	0.9600
N5—N6	1.370 (4)	C20—H20C	0.9600
N6—C14	1.354 (4)	C21—C22	1.480 (5)
N6—B1	1.547 (5)	C22—C23	1.381 (5)
N7—C17	1.343 (4)	C23—C24	1.373 (5)
N7—N8	1.359 (3)	C23—H23	0.9300
N8—C19	1.350 (4)	C24—C25	1.483 (5)
N8—B2	1.551 (5)	C25—H25A	0.9600
N9—C22	1.338 (4)	C25—H25B	0.9600
N9—N10	1.358 (3)	C25—H25C	0.9600
N10—C24	1.347 (4)	B1—H1B	1.11 (3)
N10—B2	1.546 (5)	B2—H21B	1.16 (3)
C1—C2	1.471 (5)	B2—H22B	1.08 (4)
			
N7—Co1—H21B	72.2 (9)	C9—C10—H10A	109.5
N7—Co1—N9	93.97 (10)	C9—C10—H10B	109.5
H21B—Co1—N9	73.4 (9)	H10A—C10—H10B	109.5
N7—Co1—N3	99.66 (10)	C9—C10—H10C	109.5
H21B—Co1—N3	169.7 (9)	H10A—C10—H10C	109.5
N9—Co1—N3	101.63 (10)	H10B—C10—H10C	109.5
N7—Co1—N1	166.50 (10)	F9—C11—F7	106.3 (4)
H21B—Co1—N1	98.7 (9)	F9—C11—F8	106.2 (4)
N9—Co1—N1	92.81 (10)	F7—C11—F8	105.0 (4)
N3—Co1—N1	90.37 (10)	F9—C11—C12	115.0 (3)
N7—Co1—N5	89.25 (10)	F7—C11—C12	112.6 (3)
H21B—Co1—N5	93.9 (9)	F8—C11—C12	111.2 (3)
N9—Co1—N5	165.12 (10)	N5—C12—C13	111.5 (3)
N3—Co1—N5	92.12 (10)	N5—C12—C11	123.5 (3)
N1—Co1—N5	81.32 (10)	C13—C12—C11	125.0 (3)
C2—N1—N2	105.2 (3)	C14—C13—C12	105.9 (3)
C2—N1—Co1	140.2 (2)	C14—C13—H13	127.1
N2—N1—Co1	114.44 (18)	C12—C13—H13	127.1
C4—N2—N1	110.6 (3)	N6—C14—C13	107.4 (3)
C4—N2—B1	128.4 (3)	N6—C14—C15	123.2 (4)
N1—N2—B1	120.8 (2)	C13—C14—C15	129.4 (4)
C7—N3—N4	104.9 (3)	C14—C15—H15A	109.5
C7—N3—Co1	139.2 (2)	C14—C15—H15B	109.5
N4—N3—Co1	115.93 (19)	H15A—C15—H15B	109.5
C9—N4—N3	110.8 (3)	C14—C15—H15C	109.5
C9—N4—B1	129.0 (3)	H15A—C15—H15C	109.5
N3—N4—B1	120.0 (2)	H15B—C15—H15C	109.5
C12—N5—N6	104.9 (3)	F10—C16—F12	107.6 (4)
C12—N5—Co1	139.6 (2)	F10—C16—F11	107.4 (4)
N6—N5—Co1	113.09 (19)	F12—C16—F11	103.1 (3)
C14—N6—N5	110.3 (3)	F10—C16—C17	114.9 (3)
C14—N6—B1	129.7 (3)	F12—C16—C17	112.0 (3)
N5—N6—B1	119.7 (2)	F11—C16—C17	111.0 (4)
C17—N7—N8	105.5 (2)	N7—C17—C18	110.9 (3)
C17—N7—Co1	146.1 (2)	N7—C17—C16	122.6 (3)
N8—N7—Co1	108.39 (18)	C18—C17—C16	126.2 (3)
C19—N8—N7	110.2 (3)	C19—C18—C17	105.5 (3)
C19—N8—B2	134.5 (3)	C19—C18—H18	127.2
N7—N8—B2	114.9 (2)	C17—C18—H18	127.2
C22—N9—N10	105.5 (2)	N8—C19—C18	107.9 (3)
C22—N9—Co1	146.2 (2)	N8—C19—C20	122.8 (3)
N10—N9—Co1	108.18 (18)	C18—C19—C20	129.3 (3)
C24—N10—N9	111.0 (3)	C19—C20—H20A	109.5
C24—N10—B2	133.8 (3)	C19—C20—H20B	109.5
N9—N10—B2	115.2 (2)	H20A—C20—H20B	109.5
F3—C1—F2	106.9 (4)	C19—C20—H20C	109.5
F3—C1—F1	105.5 (4)	H20A—C20—H20C	109.5
F2—C1—F1	106.2 (3)	H20B—C20—H20C	109.5
F3—C1—C2	114.7 (3)	F15—C21—F13	107.5 (4)
F2—C1—C2	110.7 (3)	F15—C21—F14	104.3 (4)
F1—C1—C2	112.2 (4)	F13—C21—F14	105.2 (4)
N1—C2—C3	110.6 (3)	F15—C21—C22	112.3 (4)
N1—C2—C1	123.9 (3)	F13—C21—C22	114.2 (3)
C3—C2—C1	125.4 (3)	F14—C21—C22	112.6 (4)
C4—C3—C2	106.4 (3)	N9—C22—C23	110.6 (3)
C4—C3—H3	126.8	N9—C22—C21	122.9 (3)
C2—C3—H3	126.8	C23—C22—C21	126.4 (3)
C3—C4—N2	107.1 (3)	C24—C23—C22	105.8 (3)
C3—C4—C5	129.1 (3)	C24—C23—H23	127.1
N2—C4—C5	123.8 (3)	C22—C23—H23	127.1
C4—C5—H5A	109.5	N10—C24—C23	107.1 (3)
C4—C5—H5B	109.5	N10—C24—C25	123.0 (3)
H5A—C5—H5B	109.5	C23—C24—C25	129.9 (3)
C4—C5—H5C	109.5	C24—C25—H25A	109.5
H5A—C5—H5C	109.5	C24—C25—H25B	109.5
H5B—C5—H5C	109.5	H25A—C25—H25B	109.5
F4—C6—F5	108.3 (5)	C24—C25—H25C	109.5
F4—C6—F6	108.8 (5)	H25A—C25—H25C	109.5
F5—C6—F6	102.0 (4)	H25B—C25—H25C	109.5
F4—C6—C7	115.3 (4)	N4—B1—N2	110.5 (3)
F5—C6—C7	111.9 (5)	N4—B1—N6	109.8 (3)
F6—C6—C7	109.6 (5)	N2—B1—N6	109.0 (3)
N3—C7—C8	111.4 (3)	N4—B1—H1B	108.1 (18)
N3—C7—C6	124.1 (4)	N2—B1—H1B	109.6 (18)
C8—C7—C6	124.5 (4)	N6—B1—H1B	109.8 (18)
C9—C8—C7	105.6 (3)	N10—B2—N8	109.2 (3)
C9—C8—H8	127.2	N10—B2—H21B	104.2 (17)
C7—C8—H8	127.2	N8—B2—H21B	103.6 (17)
N4—C9—C8	107.4 (3)	N10—B2—H22B	110.1 (19)
N4—C9—C10	123.0 (4)	N8—B2—H22B	110.1 (19)
C8—C9—C10	129.6 (4)	H21B—B2—H22B	119 (3)
			
N7—Co1—N1—C2	−78.5 (5)	N4—N3—C7—C8	0.1 (4)
H21B—Co1—N1—C2	−32.0 (10)	Co1—N3—C7—C8	179.3 (3)
N9—Co1—N1—C2	41.6 (3)	N4—N3—C7—C6	178.6 (4)
N3—Co1—N1—C2	143.3 (3)	Co1—N3—C7—C6	−2.2 (7)
N5—Co1—N1—C2	−124.6 (3)	F4—C6—C7—N3	8.4 (7)
N7—Co1—N1—N2	97.9 (4)	F5—C6—C7—N3	−116.0 (5)
H21B—Co1—N1—N2	144.4 (9)	F6—C6—C7—N3	131.6 (5)
N9—Co1—N1—N2	−142.0 (2)	F4—C6—C7—C8	−173.3 (5)
N3—Co1—N1—N2	−40.3 (2)	F5—C6—C7—C8	62.3 (7)
N5—Co1—N1—N2	51.8 (2)	F6—C6—C7—C8	−50.1 (6)
C2—N1—N2—C4	−0.2 (3)	N3—C7—C8—C9	−0.4 (5)
Co1—N1—N2—C4	−177.8 (2)	C6—C7—C8—C9	−178.8 (4)
C2—N1—N2—B1	175.2 (3)	N3—N4—C9—C8	−0.5 (4)
Co1—N1—N2—B1	−2.4 (3)	B1—N4—C9—C8	175.0 (3)
N7—Co1—N3—C7	49.1 (4)	N3—N4—C9—C10	179.7 (4)
H21B—Co1—N3—C7	13 (5)	B1—N4—C9—C10	−4.8 (6)
N9—Co1—N3—C7	−47.0 (4)	C7—C8—C9—N4	0.5 (5)
N1—Co1—N3—C7	−140.0 (4)	C7—C8—C9—C10	−179.7 (5)
N5—Co1—N3—C7	138.7 (4)	N6—N5—C12—C13	−1.3 (4)
N7—Co1—N3—N4	−131.7 (2)	Co1—N5—C12—C13	158.4 (3)
H21B—Co1—N3—N4	−168 (5)	N6—N5—C12—C11	176.0 (3)
N9—Co1—N3—N4	132.1 (2)	Co1—N5—C12—C11	−24.4 (5)
N1—Co1—N3—N4	39.2 (2)	F9—C11—C12—N5	86.5 (5)
N5—Co1—N3—N4	−42.1 (2)	F7—C11—C12—N5	−35.4 (5)
C7—N3—N4—C9	0.3 (4)	F8—C11—C12—N5	−152.9 (3)
Co1—N3—N4—C9	−179.2 (2)	F9—C11—C12—C13	−96.6 (5)
C7—N3—N4—B1	−175.7 (3)	F7—C11—C12—C13	141.5 (4)
Co1—N3—N4—B1	4.8 (4)	F8—C11—C12—C13	24.0 (5)
N7—Co1—N5—C12	−30.5 (3)	N5—C12—C13—C14	0.5 (4)
H21B—Co1—N5—C12	41.5 (10)	C11—C12—C13—C14	−176.7 (3)
N9—Co1—N5—C12	72.2 (5)	N5—N6—C14—C13	−1.3 (4)
N3—Co1—N5—C12	−130.2 (3)	B1—N6—C14—C13	172.8 (3)
N1—Co1—N5—C12	139.8 (3)	N5—N6—C14—C15	179.5 (4)
N7—Co1—N5—N6	128.0 (2)	B1—N6—C14—C15	−6.4 (6)
H21B—Co1—N5—N6	−159.9 (9)	C12—C13—C14—N6	0.5 (4)
N9—Co1—N5—N6	−129.3 (4)	C12—C13—C14—C15	179.6 (4)
N3—Co1—N5—N6	28.4 (2)	N8—N7—C17—C18	0.3 (4)
N1—Co1—N5—N6	−61.7 (2)	Co1—N7—C17—C18	−176.5 (3)
C12—N5—N6—C14	1.6 (3)	N8—N7—C17—C16	−174.5 (3)
Co1—N5—N6—C14	−164.2 (2)	Co1—N7—C17—C16	8.7 (7)
C12—N5—N6—B1	−173.2 (3)	F10—C16—C17—N7	−0.7 (6)
Co1—N5—N6—B1	21.0 (3)	F12—C16—C17—N7	−123.9 (4)
H21B—Co1—N7—C17	−156.8 (10)	F11—C16—C17—N7	121.4 (4)
N9—Co1—N7—C17	132.1 (4)	F10—C16—C17—C18	−174.6 (4)
N3—Co1—N7—C17	29.6 (4)	F12—C16—C17—C18	62.2 (6)
N1—Co1—N7—C17	−107.9 (5)	F11—C16—C17—C18	−52.5 (5)
N5—Co1—N7—C17	−62.4 (4)	N7—C17—C18—C19	0.1 (4)
H21B—Co1—N7—N8	26.5 (10)	C16—C17—C18—C19	174.6 (4)
N9—Co1—N7—N8	−44.6 (2)	N7—N8—C19—C18	0.6 (4)
N3—Co1—N7—N8	−147.1 (2)	B2—N8—C19—C18	−171.6 (4)
N1—Co1—N7—N8	75.4 (5)	N7—N8—C19—C20	179.7 (4)
N5—Co1—N7—N8	120.8 (2)	B2—N8—C19—C20	7.5 (6)
C17—N7—N8—C19	−0.5 (4)	C17—C18—C19—N8	−0.4 (4)
Co1—N7—N8—C19	177.6 (2)	C17—C18—C19—C20	−179.5 (4)
C17—N7—N8—B2	173.4 (3)	N10—N9—C22—C23	−0.1 (4)
Co1—N7—N8—B2	−8.5 (3)	Co1—N9—C22—C23	−175.9 (3)
N7—Co1—N9—C22	−134.0 (4)	N10—N9—C22—C21	178.1 (3)
H21B—Co1—N9—C22	155.9 (10)	Co1—N9—C22—C21	2.3 (6)
N3—Co1—N9—C22	−33.3 (4)	F15—C21—C22—N9	−137.6 (4)
N1—Co1—N9—C22	57.6 (4)	F13—C21—C22—N9	−14.9 (6)
N5—Co1—N9—C22	123.8 (5)	F14—C21—C22—N9	105.1 (4)
N7—Co1—N9—N10	50.21 (19)	F15—C21—C22—C23	40.3 (6)
H21B—Co1—N9—N10	−19.8 (10)	F13—C21—C22—C23	163.0 (4)
N3—Co1—N9—N10	150.93 (18)	F14—C21—C22—C23	−77.1 (5)
N1—Co1—N9—N10	−118.11 (19)	N9—C22—C23—C24	0.6 (4)
N5—Co1—N9—N10	−51.9 (5)	C21—C22—C23—C24	−177.5 (4)
C22—N9—N10—C24	−0.5 (3)	N9—N10—C24—C23	0.8 (4)
Co1—N9—N10—C24	177.1 (2)	B2—N10—C24—C23	178.0 (3)
C22—N9—N10—B2	−178.2 (3)	N9—N10—C24—C25	−177.5 (3)
Co1—N9—N10—B2	−0.6 (3)	B2—N10—C24—C25	−0.3 (6)
N2—N1—C2—C3	−0.1 (4)	C22—C23—C24—N10	−0.8 (4)
Co1—N1—C2—C3	176.5 (3)	C22—C23—C24—C25	177.3 (4)
N2—N1—C2—C1	178.3 (3)	C9—N4—B1—N2	121.5 (4)
Co1—N1—C2—C1	−5.1 (6)	N3—N4—B1—N2	−63.3 (4)
F3—C1—C2—N1	34.6 (5)	C9—N4—B1—N6	−118.2 (4)
F2—C1—C2—N1	155.7 (4)	N3—N4—B1—N6	57.0 (4)
F1—C1—C2—N1	−85.9 (5)	C4—N2—B1—N4	−124.1 (3)
F3—C1—C2—C3	−147.2 (4)	N1—N2—B1—N4	61.4 (4)
F2—C1—C2—C3	−26.2 (6)	C4—N2—B1—N6	115.1 (3)
F1—C1—C2—C3	92.3 (5)	N1—N2—B1—N6	−59.4 (4)
N1—C2—C3—C4	0.3 (4)	C14—N6—B1—N4	112.3 (4)
C1—C2—C3—C4	−178.1 (4)	N5—N6—B1—N4	−74.1 (3)
C2—C3—C4—N2	−0.4 (4)	C14—N6—B1—N2	−126.5 (3)
C2—C3—C4—C5	178.7 (4)	N5—N6—B1—N2	47.1 (4)
N1—N2—C4—C3	0.3 (4)	C24—N10—B2—N8	111.9 (4)
B1—N2—C4—C3	−174.6 (3)	N9—N10—B2—N8	−71.0 (3)
N1—N2—C4—C5	−178.7 (4)	C19—N8—B2—N10	−109.7 (4)
B1—N2—C4—C5	6.3 (6)	N7—N8—B2—N10	78.3 (3)

### Table 2 Flourine interaction geometry (Å, °)

Cg1, Cg2, Cg3 and Cg4 are the centroids of the N3/N4/C9/C8/C7, N5/N6/C14/C13/C12, N7/N8/C19/C18/C17 and N9/N10/C24/C23/C22 rings, respectively.

**Table:**

C—F···Cg	C—F	F···Cg	C···Cg	C—F···Cg
C1—F1···Cg4	1.333 (5)	3.319 (4)	3.798 (5)	100.7 (3)
C1—F2···Cg2^i^	1.331 (4)	3.341 (3)	4.565 (4)	152.6 (2)
C1—F3···Cg4	1.316 (5)	3.273 (3)	3.798 (5)	103.3 (3)
C11—F9···Cg3	1.295 (5)	3.025 (4)	3.826 (4)	119.1 (3)
C16—F10···Cg1	1.298 (5)	3.252 (4)	4.279 (4)	135.8 (3)
C16—F10···Cg2	1.298 (5)	3.208 (3)	4.061 (4)	122.8 (2)
C16—F11···Cg4^ii^	1.324 (5)	3.605 (4)	4.467 (4)	123.0 (3)
C21—F13···Cg1	1.321 (5)	3.373 (3)	4.346 (5)	130.3 (3)

Symmetry codes: (i) 1/2-x, 3/2-y, -z; (ii) 1/2-x, 1/2+y, 1/2-z.
